# Essential Oil Variability in Iranian Populations of *Heracleum persicum* Desf. ex Fischer: A Rich Source of Hexyl Butyrate and Octyl Acetate

**DOI:** 10.3390/molecules27196296

**Published:** 2022-09-23

**Authors:** Seyed Hamid Mustafavi, Amin Abbasi, Mohammad Reza Morshedloo, Mirian Pateiro, Jose M. Lorenzo

**Affiliations:** 1 Karaneh Nilgoon Ofogh (KNO) Agricultural Company, Tehran 1631668951, Iran; 2 Department of Plant Production and Genetics, Faculty of Agriculture, Maragheh University, Maragheh 8311155181, Iran; 3 Department of Horticultural Science, Faculty of Agriculture, University of Maragheh, Maragheh 8311155181, Iran; 4 Centro Tecnológico de la Carne de Galicia, Rúa Galicia N° 4, Parque Tecnológico de Galicia, 32900 San Cibrao das Viñas, Ourense, Spain; 5 Área de Tecnoloxía dos Alimentos, Facultade de Ciencias, Universidade de Vigo, 32004 Ourense, Ourense, Spain

**Keywords:** phytochemical diversity, chemotype, hexyl butyrate, octyl acetate

## Abstract

*Heracleum persicum* Desf. ex Fischer seeds are a rich source of essential oils (EOs) with high antimicrobial and antioxidant effects. In order to determine the phytochemical variability in various Iranian *H. persicum* populations, seed samples were collected from 10 different climatic locations. The current study indicated that hexyl butyrate (20.9–44.7%), octyl acetate (11.2–20.3%), hexyl-2-methylbutyrate (4.81–8.64%), and octyl 2-methyl butyrate (3.41–8.91%) were the major components of the EOs. The maximum (44.7%) and the minimum (20.9%) content of hexyl butyrate were obtained from Kaleibar and Sari populations, respectively. Moreover, the octyl acetate content ranged from 2% (in Mahdasht) to 20.3% in Torghabeh population. The CA and PCA analysis divided the 10 Iranian *H. persicum* populations into three major groups. Populations from Khanghah, Kaleibar, Shebeilo, Showt, Mahdasht, and Amin Abbad showed a distinct separation in comparison with the other populations, having high contents of hexyl butyrate (39.8%) and low contents of octyl acetate (13.5%) (Chemotype II). According to correlation analysis, the highest correlation coefficient was among habitat elevation and hexyl butyrate content. In addition, the mean annual precipitation was negatively correlated with the content of hexyl butyrate. Although octyl acetate content showed high correlation with soil EC and mean annual temperature, it was not statistically significant. In general, in order to have plants with a high content of hexyl butyrate, it is recommended to harvest these plants from regions with high altitude and low rainfall such as Kaleibar.

## 1. Introduction

The genus *Heracleum*, with about 109 species in the world and predominantly in Asia, is one of the widespread members of the Apiaceae family [1]. *Heracleum persicum* Desf. ex Fischer is an aromatic spice, which is known as Golpar in Iran. Seeds and shoots of *H. persicum* are traditionally used as a flavoring agent and spice in food products. Furthermore, the leaves and fruits of the plant are used as carminative, antiseptic, analgesic, and digestive agents in Iranian traditional medicine [2,3,4]. Food microbial contamination is one of the most important unfavorable factors, which not only causes food deterioration but also induces many diseases. It has been reported that as many as 30% of people in industrialized countries suffer from food-borne diseases each year. Although application of synthetic antioxidants could control microbial contamination in foods, their application is restricted due to carcinogenicity. Thus, finding natural, safe, and effective antioxidants and antimicrobials has gained more attention for controlling food-borne pathogens [5,6,7,8,9,10,11]. Essential oils (EOs) are receiving a good deal of attention for use as food preservatives to reduce the oxidative reactions during food processing and storage [11]. *H. persicum* seeds are a rich source of EOs, which could be used as antibacterial and antioxidant agents in food and pharmaceutical industries. Mostaghim et al. [12] reported that the EO of *H. persicum* had potential for use in food industry to inhibit pathogen growth and improve food quality and safety. They reported that the main constituents of seed EO of *H. persicum* were hexyl butyrate (35.5%) and octyl acetate (27%). Hexyl butyrate is a fatty acid ester obtained from the formal condensation of hexanol with butyric acid. The aliphatic esters octyl acetate is formed from 1-octanol (octyl alcohol) and acetic acid. Hexyl esters, most notably hexyl butyrate, are widely used as fragrance and flavor in food and pharmaceutical industries [13]. In addition, Maggi et al. [14] showed that antioxidant and anticancer activities of hogweed (*Heracleum sphondylium* L. subsp. *ternatum* (Velen.) Brummitt) EO mainly depend on octyl acetate and octyl butyrate.

EO content, constituents, and biological effects can be significantly influenced by different exogenous and endogenous factors [11]. The exogenous factors are factors regulated by environmental conditions such as precipitation, light, growing site, and soil. In contrast, the endogenous factors are genetically related factors. Previous studies revealed that climatic conditions and soil properties influence the phytochemicals and antioxidant properties of aromatic plants [15]. For instance, Vokou et al. [16] reported that the higher content of EO in *Origanum vulgare* ssp. *hirtum* was achieved in higher altitude.

Thus, a comparative analysis of EO quantity and quality collected from various geographical locations would be vital for industrial purposes. Moreover, existing variation could be used in breeding programs for the development of cultivars satisfied levels of active ingredients, which in turn may promote the cultivation of this high value aromatic and spice plant [16]. The objective of the present study was to evaluate the phytochemical diversity of Iranian wild populations of *H. persicum*, which is important for the development of in situ and ex situ conservation programs and for the selection of preferable populations for breeding programs and industries.

## 2. Results and Discussion

### 2.1. EO Content

**Figure 1:**
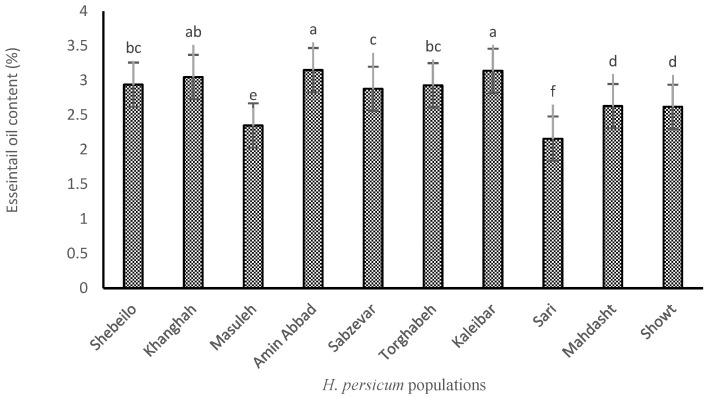
EO content of different *H. persicum* populations. Means ± standard deviations with same letters do not have significant difference from each other.

Analysis of variance showed that there were significant differences among *H. persicum* populations for EO content. Although the highest EO content (3.15%) was obtained from the plants collected from Amin Abbad, there were no statistically significant differences between samples obtained from this site and those harvested from Kaleibar (3.14%) and Khanghah (3.05%). The lowest EO content (2.16%) was found in the samples related to Sari. As shown in Figure 1, the highest content of EO is related to the plants originated from regions with higher altitude and harsher environmental conditions. Also, plants grown in regions with lower altitude and more suitable temperature and rainfall conditions, such as Sari, have a lower percentage of EO, which indicates the moderating effect of EO in dealing with environmental stress conditions. In fact, EO production was involved in protection against changing environments and stressful constraints during growth. Similar results were also reported by Mirza and Najafpour [17] and Radjabian et al. [18], both on *Heracleum gorganicum* Rech.f.

### 2.2. EO Components Analysis

A total of 23 components were identified in the EOs representing 86.5–99.3% of the total identified components (Table 1). The GC−MS analysis revealed that hexyl butyrate (20.9–44.7%), octyl acetate (11.2–20.3%), hexyl-2-methylbutyrate (4.81–8.64%) and octyl 2-methyl butyrate (3.41–8.91%) were the major components, while butyl butyrate (0.82–2.22%), butyl 2-methylbutyrate (0.74–2.12%), hexyl isobutyrate (3.06–6.43%), n-decanal (0.36–3.14%), octyl isobutyrate (1.85–5.85%), n-hexyl hexanoate (3.32–7.98%), and n-octyl butyrate (2.33–5.65%) had lower contents in the EO of *H. persicum*.

The maximum (44.7%) and minimum (20.9%) contents of hexyl butyrate were obtained from Kaleibar and Sari populations, respectively (Table 1). Moreover, the octyl acetate content ranged from 11.2% (in Mahdasht) to 20.3% in Torghabeh population. However, Masuleh population showed the highest contents of octyl isobutyrate (5.85%), n-hexyl hexanoate (7.98%), and n-octyl butyrate (5.65%) (Table 1).

The highest content of isopropyl 2-methylbutyrate (2.25%) and linalool (1.32%) were related to Sari population. Also, the samples collected from the Showt region showed the highest contents of isopropyl isovalerate, isobutyl 2-methylbutyrate, hexyl acetate, and limonene. Butyl butyrate content ranged from 1.1% to 2.4% in Sari and Torghabeh populations, respectively. The highest content of o-cymene (2.5%), γ-terpinene (0.81%), n-decanal (2.61%), and hexyl-2-methylbutyrate (8.64%) were obtained from Amin Abbad population.

The EO composition of *H. persicum* having different origins has been previously reported. The main constituents in some reports were hexyl butanoate (28.32%), heptyl isobutanoate (24.05%), hexyl 2-methylbutanoate (8.24%) [19] and octyl acetate and hexyl butyrate [20,21]. Our results revealed that EO content and composition of *H. persicum* significantly varied among the different populations which indicates the adaptability of the plant to the ecological conditions of the habitat. Sangwan et al. [22] demonstrated that genetics and ecological factors are two key factors influencing the EO variability. For example, Pouyanfar et al. [23] indicated that the higher amount of rainfall increases the caryophyllene oxide and bergamotol acetate contents in *Melissa officinalis* L. Diversity in plant active ingredients in response to ecological factors has been previously reported in Apiaceae [24] and Asteraceae families [25].

### 2.3. Classification of the H. persicum Populations

To determine the phytochemical variability and identify the different chemotypes of *H. persicum*, their EO components were submitted to principal component (PCA) and cluster analysis (CA). According to PCA, the first four components of the PCA explained 77.6% of the total variation (Table 2). The first PC (PC1) showed 26% of the total variation and had a positive correlation with isopropyl 2-methylbutyrate (0.71), hexyl-2-methylbutyrate (0.71), octyl isobutyrate (0.76%), n-hexyl hexanoate (0.56%) and n-octyl butyrate (0.73%). The second component (PC2) was formed of o-cymene, limonene, butyl 2-methylbutyrate, hexyl isobutyrate, octyl acetate, and hexyl isovalerate compounds. The third PC (PC3) had positive correlation with isobutyl butyrate and butyl butyrate, hexyl acetate, and n-decanal and a negative correlation with octyl 2-methyl butyrate. Finally, the fourth PC showed a positive and negative correlation with linalool and thymol, respectively (Table 2).

**Figure 2:**
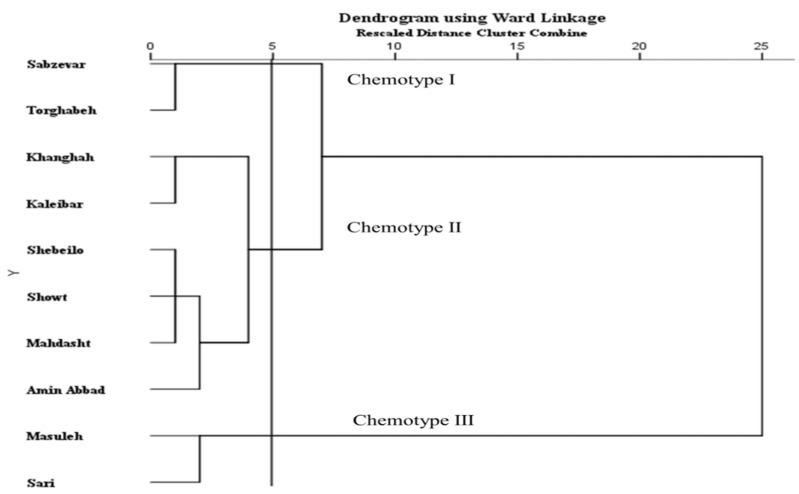
Cluster analysis of 10 populations of *H. persicum* based on EO components. Populations were clustered using Ward Linkage algorithm.

**Figure 3:**
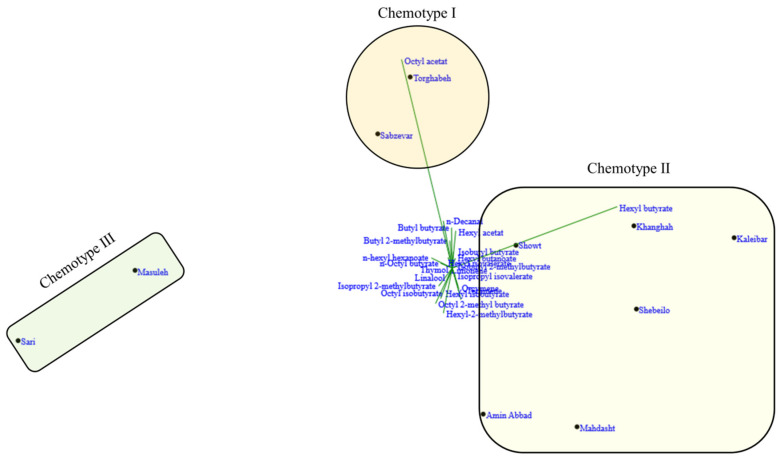
PCA biplot analysis of *H. persicum* populations based on phytochemical traits.

The biplots for PCA and dendrogram for cluster analysis showed similar patterns for populations grouping. The CA and PCA analysis divided the 10 Iranian *H. persicum* populations into three major groups, each representing a distinct chemotype. According to Figure 2 and Figure 3 and Table 1, chemotype I was made of two populations (including Sabzevar and Torghabeh), with hexyl butyrate (34.6%) and octyl acetate (19.6%) as the major compound of the EO. Populations from Khanghah, Kaleibar, Shebeilo, Showt, Mahdasht, and Amin Abbad showed a distinct separation in comparison with the other populations, having high contents of hexyl butyrate (39.8%) and low content of octyl acetate (13.5%) (Chemotype II). Chemotype III was comprised of Masuleh and Sari with isopropyl 2-methylbutyrate, octyl isobutyrate, and n-hexyl hexanoate (Table 1). Despite the fact that geographical and climatic conditions influence the distribution of populations into different chemotypes, it seems to be associated with the local selective forces. Hanover [26] reported that the appearance of chemotypes is strongly controlled by genetic factors. The results illustrated that geographic and ecological factors had a key role in the occurrence of different chemotypes such that populations with close and similar geographic distances may be subject to different chemotypes due to forces of ecological conditions. 

### 2.4. Environmental Factors Affecting the Chemical Variability

**Figure 4:**
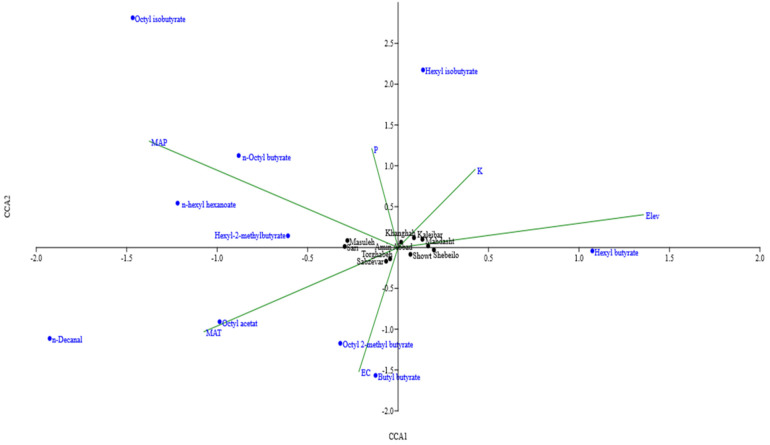
Canonical correspondence analysis biplot of *H. persicum* populations, linking percentages of the major components, collected site and their climatic characteristics.

Canonical correspondence analysis (CCA) was carried out according to matrix linking percentages of ten major constituents and six environmental factors to assess the environmental factors impact on chemical variability (Figure 4). The first CCA (CC1) variable in relation with collected sites and their climatic characteristics revealed that soil K content and altitude (positively) and soil EC, available P, MAP, and MAT (negatively) had a great share in the formation of this canonical variable. Coefficients of the first canonical variable in relation with major components revealed that hexyl butyrate (positively) and n-octyl butyrate, n-hexyl hexanoate, and hexyl-2-methylbutyrate (negatively) had a great share on the formation of this canonical variable. Furthermore, populations from Khanghah, Kaleibar, Shebeilo, Showt, Mahdasht, and Amin Abbad were positively, and those from Sabzevar, Torghabeh, Masuleh and Sari were negatively affected by the first canonical set (Table 3).

### 2.5. Correlation Results

Correlation analysis was also carried out to confirm the results of CCA (Table 4). The octyl acetate content showed a positive and significant correlation with butyl butyrate and n-decanal contents. Although n-hexyl hexanoate content was positively correlated with n-octyl butyrate, a negative and significant correlation was found with hexyl butyrate content. In addition, the content of n-octyl butyrate was positively correlated with the octyl isobutyrate content. There was no significant coefficient between hexyl isobutyrate, hexyl-2-methylbutyrate and octyl 2-methyl butyrate contents with the other oil components. In the case of environmental factors and soil characteristics, the highest correlation coefficient was among habitat elevation and hexyl butyrate content. In addition, soil available P and EC positively correlated with hexyl isobutyrate and butyl butyrate contents, respectively. Other positive correlations were between mean annual temperature and octyl 2-methyl butyrate content. In addition, mean annual precipitation was negatively correlated with the content of hexyl butyrate, but a positive and significant correlation was found with octyl isobutyrate, n-hexyl hexanoate, and n-octyl butyrate contents. Maggi et al. [14] revealed that the cultivated varieties of *Heracleum sphondylium* had lower contents of octyl acetate than wild populations.

## 3. Materials and Methods

### 3.1. Plant Materials

**Figure 5:**
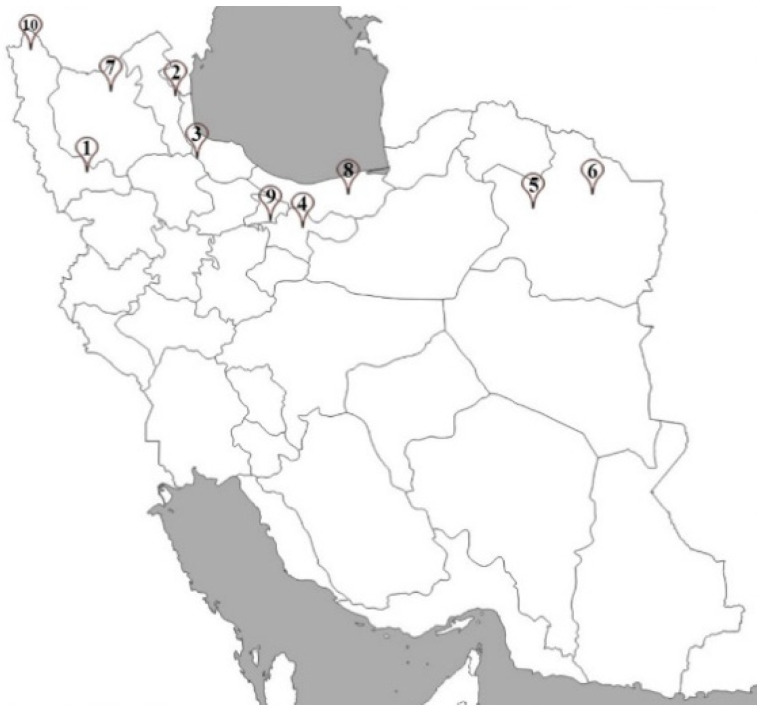
Sample collection sites located in different provinces of Iran. Numbers showing the collection sites.

The seeds of *H. persicum* wild populations were collected from 10 different geographical regions in Iran (Figure 5). Geographic coordinates, including altitude, latitude, and longitude of each region were recorded using Global Positioning System (GPS) and are presented in Table 5. In each studied site, the soil samples were taken from the root depth of the plants (0–30 cm) and were analyzed to determine the main physicochemical properties of the soil, including available phosphorus, potassium, and electrical conductivity (EC) (Table 5) [27]. Sampling was done using a random–systematic method along with the located transects. The plant seeds were collected at ripening stage. They were identified and recorded by Seed Bank of Medicinal Plants Institute, ACECR with the HP-436 code.

### 3.2. Preparation of the EO

To extract the EO, the seed samples of *H. persicum* in three replications were ground to obtain a fine powder. Then, the EOs were extracted by hydro-distillation method for 3 h using a Clevenger-type apparatus. The EOs were dried over anhydrous sodium sulphate and kept at 4 °C until the analysis stage. After that, EO content (%) was measured based on mass of EOs (g) extracted from 40 g mass of seed samples using the following formula:

EO content (%) = [mass of EO (g)/mass of seed dry matter (g)] × 100

### 3.3. GC-MS Analysis

#### 3.3.1. Gas Chromatography (GC)

Gas chromatography analysis was performed using a Younglin Acme6000 gas chromatograph equipped with BP-5 column (30 m, 0.25 mm internal diameter, and 0.25 µm film thickness). The column temperature program was set as follows: oven temperature was held at 50 °C for 5 min, from 50 to 240 °C at a rate of 3 °C per min, and to 300 °C at a rate of 15 °C per min and held at this temperature for 3 min. Injection and detection temperature was set at 290 and 300 °C, respectively. Helium was used as carrier gas with a linear velocity of 0.8 mL per min. All samples before injection were diluted in n-hexane (1:100). One microliter of the diluted sample was injected manually in the split mode 1:25 to the instrument. C8–C40 alkanes calibration std (Supelco, Bellefonte, PA, USA) was used to calculate the retention indices (RI). Identification of the individual copmounds of EOs was done by comparing their retention index (RI) with those of a computer library (Wiley, Adams and NIST05).

#### 3.3.2. Gas Chromatography/Mass Spectroscopy (GC-MS)

GC-MS analyses were carried out with an Agilent 6890 system equipped with a mass spectrometer (Agilent 5973) detector and a BPX5 column (30 m, 0.25 mm internal diameter, and 0.25 µm film thicknesses). The carrier gas was helium at a flow rate of 0.5 mL per min. The column temperature was held at 50 °C for 5 min and was gradually increased from 50 to 240 °C with a rate of 3 °C per min and to 300 °C with a rate of 15 °C per min and held at this temperature for 3 min. For detection, an electron ionization system was used with ionization energy of 70 electron Volts. The injector and ionization source temperature were 290 and 220 °C, respectively [28]. 

### 3.4. Data Analysis

Analysis of variance appropriate to the experimental design was done using SAS software (ver. 9.1). Mean comparison of the traits was done using Duncan multiple range tests at *p* ≤ 0.05 significance level. The simple Pearson correlation coefficients were calculated to determine the relationships between the studied traits. Cluster analysis, based on EO components, was performed using the ward method. In order to determine the most variable characters among the populations, factor analysis using the principal component analysis (PCA) method was performed. 

## 4. Conclusions

Our results showed that there were significant differences among *H. persicum* populations for EO content. Although the highest EO content was obtained from the plants collected from the Amin Abbad, there were no statistically significant differences between samples from this region and those harvested from Kaleibar and Khanghah. The current study indicated that the maximum and the minimum contents of hexyl butyrate were obtained from Kaleibar and Sari populations, respectively. Moreover, the octyl acetate content ranged from 11.2% (in Mahdasht) to 20.3% in Torghabeh population. The CA and PCA analysis divided the 10 Iranian *H. persicum* populations into three major groups. Populations from Khanghah, Kaleibar, Shebeilo, Showt, Mahdasht, and Amin Abbad showed a distinct separation in comparison to the other populations, having high contents of hexyl butyrate and low content of octyl acetate (Chemotype II). According to correlation analysis, the highest correlation coefficient was among habitat elevation and hexyl butyrate content. In addition, the mean annual precipitation was negatively correlated with the content of hexyl butyrate. Although octyl acetate content showed high correlation with soil EC and mean annual temperature, it was not statistically significant. In general, in order to have plants with a high content of hexyl butyrate, it is recommended to harvest these plants from regions with high altitude and low rainfall, such as Kaleibar.

## Figures and Tables

**Table 1:** The EO composition of the populations of *H. persicum*.

	KI^a^	KI^C^	Chemotexype I		Mean	Chemotype II						Mean	Chemotype III		Mean	
			Sabzevar	Torghabeh		Khanghah	Kaleibar	Shebeilo	Showt	Mahdasht	Amin Abbad		Masuleh	Sari		
Isopropyl isovalerate	874	870	0.15	0.22	0.18	0.33	0	0.53	0.87	0.85	0.22	0.46	0.11	0	0.05	ns
Isopropyl 2-methylbutyrate	960	959	0.83	0.88	0.85 b	0.59	0.32	0.78	0.95	0.62	1.61	0.81 b	2.01	2.25	2.13 a	**
Isobutyl 2-methylbutyrate	985	983	0.21	0.14	0.17	0.52	0	0.67	0.82	0.19	0.14	0.39	0	0	0	ns
Butyl butyrate	993	990	2.22	2.4	2.31	1.89	1.24	1.72	1.48	0.82	1.2	1.39	1.77	1.1	1.43	ns
Isobutyl butyrate	994	994	0.12	0.89	0.50	0.21	0.12	0.27	0.33	0	0.13	0.17	0	0	0	ns
Hexyl acetate	1007	1006	1.34	1.23	1.28	1.31	1.35	0.61	1.37	0.11	0.41	0.86	0.72	0.35	0.53	ns
O-cymene	1022	1021	0.15	0.32	0.23	1.35	1.23	0.81	0.74	0	2.5	1.11	0.28	0.37	0.32	ns
Limonene	1024	1022	0.36	0.21	0.28	0.25	0	0.35	0.44	0.24	0.02	0.21	0.22	0.34	0.28	ns
Butyl 2-methylbutyrate	1033	1033	2.12	1.85	1.98	1.39	1.14	1.91	1.47	1.02	0.74	1.27	1.53	1.51	1.52	ns
γ.-Terpinene	1054	1051	0.14	0	0.07	0.71	1.52	0.28	0.31	0.81	0.81	0.74	0.12	0.5	0.31	ns
Linalool	1095	1093	0.1	0.24	0.17	0.32	0.24	0.31	0.41	0.34	0.27	0.31	0.08	1.32	0.7	ns
Hexyl isobutyrate	1147	1150	3.06	4.13	3.59	7.8	6.43	3.05	3.89	5.62	5.35	5.35	5.08	4.87	4.97	ns
Hexyl butanoate	1191	1190	1.11	0.93	1.02	1.29	0.93	1.51	1.32	1.04	0.11	1.03	0.22	1.22	0.72	ns
n-Decanal	1201	1201	3.14	2.78	2.96	1.42	2.62	0.36	1.95	0.27	2.61	1.53	2.35	2.3	2.32	ns
Octyl acetate	1211	1208	19	20.3	19.65 a	15.2	13.9	12.7	15.6	11.2	12.5	13.51 b	18.9	18.6	18.75 a	**
Hexyl-2-methylbutyrate	1236	1239	5.78	4.95	5.36	4.84	5.9	5.14	5.75	4.81	8.64	5.84	6.17	6.71	6.44	ns
Hexyl butyrate	1238	1240	33.9	35.3	34.6 a	41.6	44.7	40.9	37.6	38.6	35.6	39.83 a	25.8	20.9	23.35 b	**
Hexyl isovalerate	1241	1243	1.16	0.87	1.01 a	0.47	0.42	0.42	0.84	0.46	0.62	0.53 b	0.63	1.04	0.83 ab	*
Thymol	1289	1287	0.39	0.36	0.37	0.13	0	0.22	0.11	0.34	0.57	0.22	0.43	0	0.21	ns
Octyl isobutyrate	1330	1329	1.85	2.02	1.93 b	3.66	4.21	2.87	2.34	2.3	3.42	3.13 b	5.85	4.32	5.08 a	*
n-Octyl butyrate	1372	1376	3.42	3.85	3.63	4.91	3.34	2.33	3.24	3.23	4.13	3.53	5.65	4.14	4.85	ns
n-hexyl hexanoate	1382	1380	4.9	4.31	4.61 b	4.75	3.43	3.68	3.32	4.31	3.7	3.86 b	7.98	4.91	6.44 a	*
Octyl 2-methyl butyrate	1436	1440	6.5	6.21	6.35	4.42	3.41	5.09	6.78	8.91	5.22	5.63	6.85	6.84	6.84	ns

KI^a^, Kovats indices taken from Adams. KI^C^, Kovats indices on DB-5 MS column, experimentally determined using homologue series of n-alkanes. ns: non-significant; * and **, significant difference at 5% and 1%, respectively.

**Table 2:** Eigenvectors of the first four principal component axes from PCA analysis of variables in studied *H. persicum* populations.

Traits	Components			
	1	2	3	4
Isopropyl 2-methylbutyrate	0.719	0.445	−0.384	0.031
Isopropyl isovalerate	−0.652	−0.233	−0.478	−0.370
Hexyl butanoate	−0.811	0.063	−0.195	0.521
Isobutyl butyrate	−0.453	0.281	0.502	−0.145
Butyl butyrate	−0.281	0.629	0.653	−0.139
Isobutyl 2-methylbutyrate	−0.760	−0.099	−0.085	−0.054
Hexyl acetate	−0.368	0.185	0.776	0.263
O-cymene	0.293	−0.628	0.364	−0.084
Limonene	−0.553	0.595	−0.443	0.143
Butyl 2-methylbutyrate	−0.465	0.774	0.180	0.125
γ-Terpinene	0.202	−0.867	0.083	0.326
Linalool	0.173	0.118	−0.560	0.665
Hexyl isobutyrate	0.370	−0.594	0.155	0.283
Hexyl butyrate	−0.585	−0.678	0.410	−0.121
n-Decanal	0.454	0.420	0.601	0.117
Octyl acetate	0.185	0.893	0.301	0.220
Hexyl-2-methylbutyrate	0.708	−0.130	−0.005	−0.138
Hexyl isovalerate	0.049	0.806	−0.014	0.145
Thymol	0.272	0.157	0.093	−0.922
Octyl isobutyrate	0.764	−0.082	−0.028	0.288
n-hexyl hexanoate	0.558	0.520	−0.082	−0.130
n-Octyl butyrate	0.728	0.235	0.166	0.003
Octyl 2-methyl butyrate	−0.041	0.407	−0.723	−0.362
% of Variance	26.084	25.500	15.628	10.399
Cumulative %	26.084	51.584	67.212	77.612

**Table 3:** Canonical coefficients, eigen-values, estimated and cumulative variance for four CCA sets between phytochemicals with environmental factors.

	CCA Sets of Environmental			
Traits	1	2	3	4
**Phytochemical**				
Butyl butyrate	−0.12	−1.56	−1.47	−1.86
Hexyl isobutyrate	0.13	2.17	−0.13	−1.26
Hexyl butyrate	1.07	−0.04	−0.16	0.03
n-Decanal	−1.92	−1.11	−3.91	0.87
Octyl acetate	−0.98	−0.91	−0.08	−0.77
Hexyl−2-methylbutyrate	−0.61	0.14	−0.54	2.92
Octyl isobutyrate	−1.46	2.81	0.59	0.71
n-hexyl hexanoate	−1.21	0.54	0.93	−0.82
n-Octyl butyrate	−0.88	1.12	−0.25	−0.44
Octyl 2-methyl butyrate	−0.31	−1.17	2.62	0.75
**Populations**				
Shebeilo	0.19	−0.02	0.04	0.02
Khanghah	0.08	0.12	−0.03	−0.09
Masuleh	−0.28	0.08	0.06	−0.04
Amin Abbad	0.02	0.06	−0.07	0.14
Sabzevar	−0.06	−0.16	−0.05	−0.02
Torghabeh	−0.04	−0.13	−0.05	−0.07
Kaleibar	0.13	0.11	−0.12	0.01
Sari	−0.29	0.01	0.05	0.02
Mahdasht	0.16	0.019	0.18	0.02
Showt	0.06	−0.08	0.01	0.03
**Environmental factors**				
Mean annual precipitation (M.A.P)	−0.68	0.65	0.17	0.01
Mean annual temperature (M.A.T)	−0.53	−0.51	0.47	−0.32
Elevation (Elev.)	0.67	0.21	−0.39	0.08
**Soil characteristics**				
Available P (ppm)	−0.07	0.61	−0.11	−0.38
Available K (ppm)	0.21	0.47	0.05	0.17
EC (dS/m)	−0.11	−0.75	−0.41	−0.24
Eigenvalues	0.022	0.008	0.005	0.003
%	55.1	21.54	14.35	7.80

**Table 4:** Correlation of major components of *Heracleum persicum* and environmental factors.

		1	2	3	4	5	6	7	8	9	10	11	12	13	14	15	16
1	Butyl butyrate	1															
2	Hexyl isobutyrate	−0.33	1														
3	Hexyl butyrate	0.02	0.24	1													
4	n-Decanal	0.38	−0.09	−0.33	1												
5	Octyl acetate	0.66 *	−0.28	−0.61	0.66 *	1											
6	Hexyl-2-methylbutyrate	−0.34	−0.005	−0.37	0.47	−0.07	1										
7	Octyl isobutyrate	−0.24	0.45	−0.41	0.12	0.11	0.33	1									
8	n-hexyl hexanoate	0.23	0.07	−0.63 *	0.17	0.52	−0.003	0.62	1								
9	n-Octyl butyrate	0.14	0.51	−0.49	0.36	0.44	0.23	0.68 *	0.77 *	1							
10	Octyl 2-methyl butyrate	−0.21	−0.33	−0.48	−0.25	0.11	−0.14	−0.24	0.3	0.007	1						
11	Elevation	0.15	0.26	0.77 **	−0.11	−0.51	0.01	−0.22	−0.34	−0.11	−0.45	1					
12	Available P	0.08	0.63 *	0.02	−0.18	0.001	−0.09	0.47	0.21	0.52	−0.44	0.03	1				
13	Available K	−0.17	0.29	0.13	−0.45	−0.42	0.18	0.22	−0.14	0.14	−0.29	0.25	0.71 *	1			
14	EC	0.68 *	−0.49	−0.01	0.55	0.52	−0.11	−0.55	−0.03	−0.18	0.02	0.04	−0.47	−0.65 *	1		
15	M.A.T	0.27	−0.52	−0.61	0.06	0.57	−0.28	−0.08	0.54	0.03	0.63 *	−0.62	−0.37	−0.58	0.46	1	
16	M.A.P	−0.29	0.38	−0.64 *	0.11	0.22	0.37	0.93 **	0.64 *	0.71 *	−0.04	−0.47	0.51	0.27	−0.55	0.05	1

*: significant at *p* ≤ 0.05: **: significant at *p* ≤ 0.01; M.A.T: mean annual temperature; M.A.P: mean annual precipitation.

**Table 5:** Origins, geographical characteristics, and soil properties of studied *H. persicum* populations.

Pop. No.	Pop. Name	Site	Latitude (N)	Longitude (E)	Altitude (m)	Available P (ppm)	Available K (ppm)	EC (dS/m)	Mean Annual Temp. [˚C]	Rainfall [mm/Year]
1	West Azerbaijan	Shebeilo	37.00191	46.13624	1298.8	15.96	432	1.56	15.4	282.2
2	Ardebil	Khanghah	38.4221	48.53571	1428.4	38.91	480	1.21	11.8	493.9
3	Gilan	Masuleh	37.15694	48.99737	928.6	17.81	280	1.12	17.9	831.3
4	Tehran	Amin Abbad	35.83881	51.56348	1706.3	12..87	420	1.78	10.8	426
5	Razavi Khorasan	Sabzevar	36.21489	57.52868	957.5	4.8	110	5.8	19.5	159.6
6	Razavi Khorasan	Torghabeh	36.30237	59.37404	1311.6	8.7	199	4.3	17.1	163.8
7	East Azerbaijan	Kaleibar	38.86724	47.01522	1353	12.02	185	1.8	11.75	418.4
8	Mazandaran	Sari	36.52126	53.03759	112.7	19.24	308	1.32	18.2	789.2
9	Alborz	Mahdasht	35.71475	50.8341	1167.8	5.29	228	1.26	17.1	243.8
10	West Azerbaijan	Showt	39.24338	44.76945	967.2	11.29	351	1.18	12.5	264.5

## Data Availability

The data used to support the findings of this study are available. Further inquiries can be directed to the corresponding authors.

## Sample Availability

Samples of the compounds are available from the authors.
